# Proceedings of First Histoplasmosis in the Americas and the Caribbean Meeting, Paramaribo, Suriname, December 4–6, 2015

**DOI:** 10.3201/eid2209.160408

**Published:** 2016-09

**Authors:** Mathieu Nacher, Antoine Adenis

**Affiliations:** Centre d’Investigation Clinique Antilles Guyane, INSERM 1424, Centre Hospitalier Andrée Rosemon, Cayenne, French Guiana; EA3593 Ecosystèmes Amazoniens et Pathologie Tropicale, Université de Guyane, Cayenne

**Keywords:** Histoplasmosis, disseminated histoplasmosis, HIV, AIDS, HIV/AIDS, amphotericin, deoxycholate, itraconazole, 80 by 20 initiative, laboratory procedures, poverty, expense, *Histoplasma* antigen, Latin America, Caribbean, the Americas, Paramaribo, Suriname

Histoplasmosis is not defined as a neglected disease, but in Latin America and the Caribbean, it is suspected that nearly 10,000 HIV-positive patients die from histoplasmosis disseminated beyond the respiratory tract yearly, placing this AIDS-defining illness among the main causes of death of HIV patients on a similar level as HIV-associated tuberculosis (*1*,*2*). During December 4–6, 2015, researchers and health professionals from 13 countries (Argentina, Brazil, Colombia, French Guiana, Guatemala, Guyana, Mexico, Panama, Surinam, Trinidad and Tobago, the United Kingdom, the United States, and Venezuela), representing organizations including the Pan American Health Organization, the US Centers for Disease Control and Prevention, the Foundation for Scientific Research of Suriname, and the Institut National de la Santé et de la Recherche Médicale, met in Paramaribo, Suriname, to define the next steps necessary to fight disseminated histoplasmosis in HIV patients (Figure).

The result of shared analysis of the situation surrounding disseminated histoplasmosis was that this disease is still rarely recognized in much of Latin America, notably among clinicians who care for HIV patients. Disseminated histoplasmosis is still often mistaken for tuberculosis and probably confounds tuberculosis statistics regarding treatment failure and mortality rates (*3*). All country representatives who attended the meeting concurred that this situation stems from the complexity and length of the diagnostic process of standard mycology and the absence of rapid, affordable, easy-to-use rapid diagnostic tests. Effects of the missed diagnoses are compounded by the lack of availability of liposomal amphotericin, the best treatment for disseminated histoplasmosis, for which attendees agreed the expense is much too high for most of Latin America (*2*). Furthermore, amphotericin B, deoxycholate, or itraconazole were sometimes unavailable in some countries. 

Disseminated histoplasmosis is often diagnosed in patients in whom HIV is diagnosed very late in the course of the HIV infection (*2*). This finding suggests that, despite improved access to antiretroviral drugs, a large number of persons with HIV remain are undiagnosed and are at risk for disseminated histoplasmosis.

Recognizing these determinants of the current situation, the researchers and health professionals who attended the meeting agreed to launch the 80 by 20 Initiative. This initiative aims to provide rapid diagnostic tests and effective treatments for disseminated histoplasmosis to 80% of the reference hospitals in Latin America and the Caribbean by the year 2020.

New rapid antigen tests are soon to be released on the market, including in Latin America and the Caribbean areas. These new tests require urine or serum samples instead of samples requiring more invasive procedures, such as bone marrow, lymph node, and liver biopsies. The tests are based on ELISA methods or lateral flow devices and thus do not require very advanced laboratory methods and are relatively inexpensive. Additionally, the meeting participants recognized that regional distributors of these tests often considerably increase the cost and that the distribution network should be chosen with care to keep prices affordable.

Access to inexpensive diagnostic tests and encouragement for screening of immunocompromised HIV-infected patients who have signs of infection is expected to generate a large increase of data related to this problem and rapidly lead to updated clinical and therapeutic algorithms for patients with advanced HIV disease, as well as an adaptation of country-specific HIV strategic plans. Of greatest consequence, a rapid diagnostic test will lead to a reduction in mortality rates, as was shown in the conference presentations by representatives of Colombia, Guatemala, and Brazil, where *Histoplasma* antigen detection in urine samples led to rapid gains in patient survival. The question of negotiating low-priced liposomal amphotericin B is in need of rapid consideration because this would affect the most severely ill patients (*4*) and is already the standard recommendation in Europe and the United States.

The 80 by 20 Initiative stems from the coalescence of shared analyses of a widespread problem through a bottom-up mechanism. However, the implementation of this initiative also requires a top-down input by national public health agencies and international health organizations. The ever-increasing circle of concerned professionals has now reached a critical mass to make this specific goal a realistic one that would save numerous lives in a cost-effective way.
